# Osteopontin (OPN/*SPP1*) isoforms collectively enhance tumor cell invasion and dissemination in esophageal adenocarcinoma

**DOI:** 10.18632/oncotarget.4161

**Published:** 2015-06-01

**Authors:** Jules Lin, Amy L. Myers, Zhuwen Wang, Derek J. Nancarrow, Daysha Ferrer-Torres, Amy Handlogten, Kimmy Leverenz, Julia Bao, Dafydd G. Thomas, Thomas D. Wang, Mark B. Orringer, Rishindra M. Reddy, Andrew C. Chang, David G. Beer, Lin Lin

**Affiliations:** ^1^ Section of Thoracic Surgery, Department of Surgery, University of Michigan, Ann Arbor, MI, USA; ^2^ Department of Pathology, University of Michigan, Ann Arbor, MI, USA; ^3^ Department of Internal Medicine, University of Michigan, Ann Arbor, MI, USA

**Keywords:** OPN/SPP1 isoforms, co-overexpression, collective function, esophageal adenocarcinoma

## Abstract

Esophageal adenocarcinoma (EAC) is often diagnosed at an advanced stage, thus understanding the molecular basis for EAC invasion and metastasis is critical. Here we report that *SPP1*/OPN was highly overexpressed in primary EACs and intracellularly localized to tumor cells. We further demonstrate that all known OPN isoforms (OPNa, b, c, 4 and 5) were frequently co-overexpressed in primary EACs. Distinct pro-invasion and dissemination phenotypes of isoform-specific OPNb and OPNc stable transfectants were observed. Expression of OPNb significantly enhanced cell migration and adhesion to laminin. In contrast, OPNc cells showed significantly decreased cell migration yet increased cell detachment. Enhanced invasion, both *in vitro* and *in vivo*, was observed for OPNb- but not OPNc-expressing cells. Inhibition of RGD integrins, one family of OPN receptors, attenuated OPNb cell migration, abrogated OPNb cell adhesion and significantly reduced OPNb cell clonogenic survival but did not affect OPNc phenotypes, indicating that OPNb but not OPNc acts through integrin-dependent signaling. Differential expression of vimentin, E-cadherin and β-catenin in OPN stable cells may account for the variation in cell adhesion and detachment between these isoforms. We conclude that while all OPN isoforms are frequently co-overexpressed in primary EACs, isoforms OPNb and OPNc enhance invasion and dissemination through collective yet distinct mechanisms.

## INTRODUCTION

The incidence of esophageal adenocarcinoma (EAC) has increased steadily in the past four decades in many western countries, including the United States, with an overall survival of only 10–19% at 5 years [1–5][http://seer.cancer.gov/statfacts/html/esoph.html]. Chronic gastroesophageal reflux disease is a major risk factor for the development of Barrett's esophagus, which leads to the increased likelihood of transformation to esophageal adenocarcinoma [6, 7]. Greater than 64% of patients with resectable EAC present with locally advanced EAC or regional nodal disease [8]. The majority of patients with resectable EAC (T2 or N1 disease or greater) are treated with neoadjuvant chemoradiation therapy followed by esophagectomy. However, at the time of resection, only 21–29% of patients show a complete response, and these complete responders have improved survival outcomes [8–10]. Overall, 80% of all cancer patients succumb to metastatic diseases [11, 12]. Controlling disease progression at presentation and enhancing patient response to existing treatment regimens are crucial to improving overall patient outcomes.

Although still poorly understood, studies have suggested that the biological cascade of invasion and metastasis includes loss of cellular adhesion, increased motility and invasiveness, entry into and survival in the circulation (intravasation), exit from the circulation at a distant site (extravasation), and colonization in the new tissue [13–16]. Mutual regulatory interactions between tumor cells and their extracellular matrix (ECM) are known to influence cancer progression [17–19]. Epithelial-mesenchymal transition (EMT) has been associated with the acquisition of motility and invasiveness and has also been reported to contribute to cancer metastasis and chemoresistance [20–24]. Osteopontin (OPN) is one of these invasion- and metastasis-associated genes [25–27].

OPN, a secreted phosphorylated extracellular matrix (ECM) protein, is encoded by the highly conserved gene *SPP1*. OPN acts through multiple adhesion receptor binding motifs, including thrombin-cleaved N-terminal integrin domains (RGD for αvβ1, αvβ3, αvβ5, α5β1 and α8β1; SVVYGLR for α4β1, α9β1, and α9β4) and a C-terminal CD44v6 domain [28–34]. Both integrin and CD44 receptors are not only involved in cell adhesion and other similar signaling pathways but also interplay with each other leading to tumor progression and metastasis [35–38]. OPN has been reported to play important roles in a wide range of biological and pathological processes, including tissue remodeling, inflammation, angiogenesis, immunity, tumor development, invasion and metastasis [33, 39]. OPN has been studied as a potential therapeutic target in the regulation of cancer metastasis [39–41]. Activation of a CD44-dependent OPN/Aurora-A/ERK1/2 pathway has been reported, and OPN-activated Aurora-A has been proposed as a potential therapeutic target in head and neck squamous cell carcinoma [42]. Moreover, expression of OPN at histologically-negative surgical margins has been associated with higher recurrence of oral squamous cell carcinomas and, therefore, may be useful as a prognostic marker of recurrence [43]. We and others have reported transcriptional overexpression of OPN/*SPP1* in primary EAC using global gene expression profiling studies [44–50]. Different OPN isoforms have been identified and have been reported to be associated with other types of malignancy [51–54]. However, whether OPN isoforms are transcriptionally exclusive in individual EACs and what their individual pathological roles are in EAC invasion and metastasis have not been fully elucidated. We report in the present study that all five OPN isoforms are co-overexpressed in the majority of primary EACs and that individual OPN isoforms show distinct phenotypes, yet act collectively in tumor invasion and dissemination in EAC/OPN cell models.

## RESULTS

### *SPP1* is highly overexpressed in primary EACs

**Figure 1:**
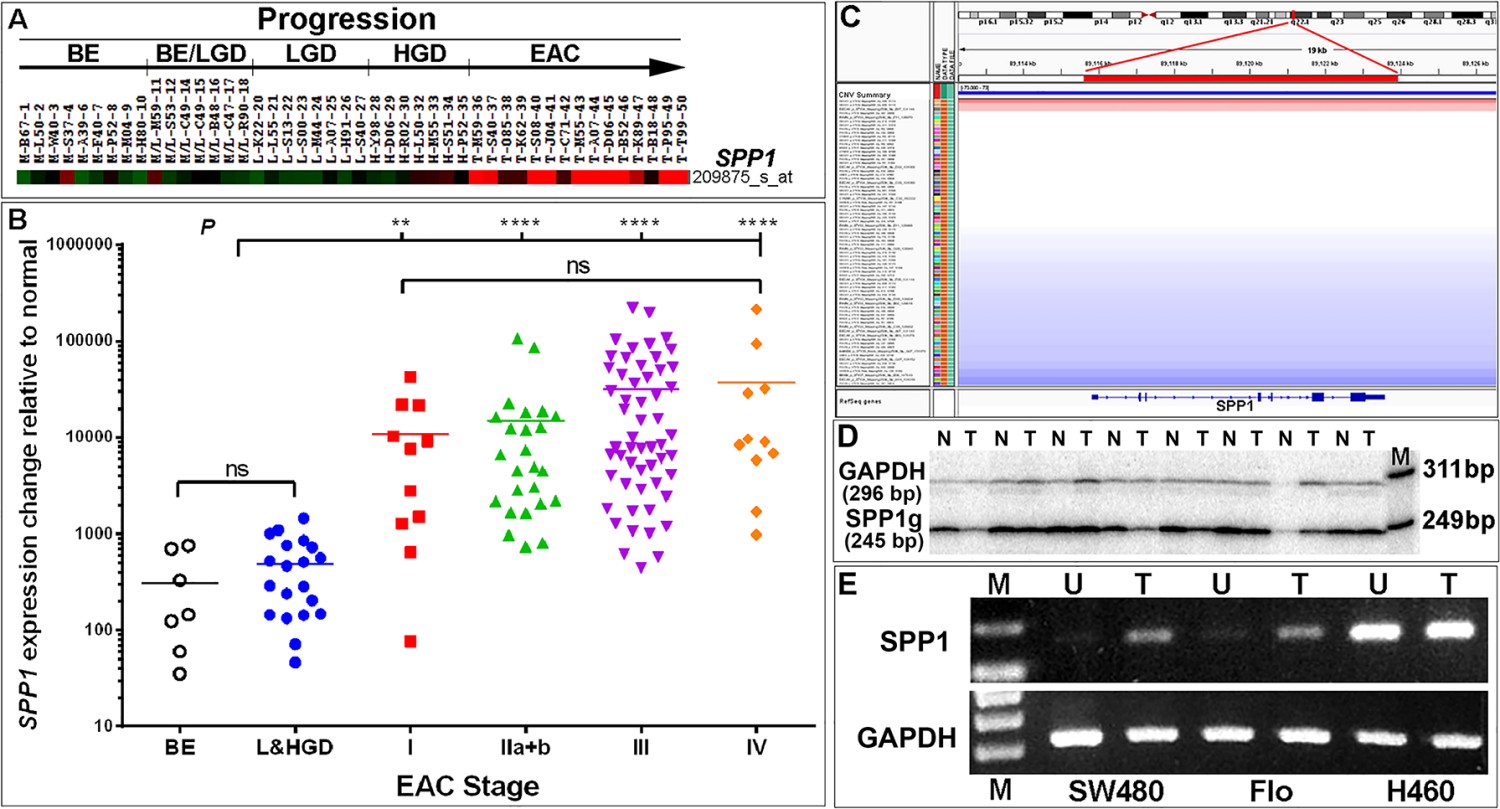
Transcriptional upregulation of *SPP1*/OPN is a frequent event in EAC patients **A.** An *SPP1*-specific heatmap indicating highly overexpressed (red) *SPP1* in EAC (*n* = 15) as compared with Barrett's esophageal metaplasia (BE) (*n* = 9), BE and low grade dysplasia (BE/LGD) (*n* = 7), LGD (*n* = 8) and high grade dysplasia (HGD) (*n* = 7, green or black) using Affymetrix U133A arrays. **B.** Overexpression of *SPP1*/OPN was validated in a cohort of 107 EACs using real-time RT-PCR analysis (Figure S1B–S1C). Significant elevated expression of *SPP1*/OPN is found in all stages of EAC as compared to premalignant Barrett's metaplasia and dysplasia samples. There was a trend towards increasing expression of *SPP1*/OPN from early to advanced stage EAC; however, the change was not statistically significant (ns, not significant, **P < 0.01, ****P < 0.0001). **C–D.** Up-regulation of *SPP1*/OPN is not due to *SPP1*/OPN gene gain or amplification as assayed using SNP arrays in 73 primary EACs (red: DNA copy number gain; blue: DNA copy number loss; gray: DNA copy number unchanged) (C). SNP analysis of the chromosomal 4q22.1 region encompassing the *SPP1*/OPN gene in 73 EACs was visualized using Integrative Genomics Viewer (IGV) software (http://www.broadinstitute.org/igv/). Unchanged copy number of the OPN locus was also confirmed by qPCR using single-tube co-amplification of *SPP1* and *GAPDH* as an internal control end-labeled with [γ-^32^P]-ATP forward primers in 86 (including the 73 EACs analyzed in SNP arrays) paired normal-EAC samples. Matched pairs of normal-EAC qPCR products (244 bp) were resolved using 8% PAGE and a representative image shown (n, normal; t, tumor; M, loading marker with 311- and 249-bp bands shown) (D). **E.** OPN expression can be regulated via epigenetic modulation. Endogenous *SPP1* levels were low in Flo and SW480 (colon carcinoma) cells but were highly abundant in H460 cells (large cell lung carcinoma) (see also Figure S2A). Cells were treated with 5-Aza-2′-deoxycytidine (decitabine) for 48 h, RNA was isolated and reverse-transcribed followed by RT-PCR using *SPP1* exon 7–8-specific primers (Table S1). PCR products were resolved on 1% agarose gels (U, untreated; T, treated with decitabine).

Affymetrix expression arrays of 46 esophageal samples representing the progression from Barrett's metaplasia and dysplasia to EAC were analyzed (GSE37200). The gene *SPP1* (secreted phosphoprotein 1, encoding osteopontin, OPN) was found to be highly overexpressed in EAC as compared to Barrett's metaplasia and dysplasia samples (Figure 1A). OPN has been reported to be associated with tumor invasion and metastasis. We validated OPN/*SPP1* overexpression in an independent cohort of 107 EAC samples using real-time RT-PCR (Figure S1) and found significantly higher expression of OPN/*SPP1* in all stages of EAC compared with Barrett's metaplasia (BE) and dysplasia (Figure 1B; *p* < 0.01 for stage I EAC and *p* < 0.0001 for all other stages). We observed a trend towards increased OPN expression among advanced stage tumors, although this did not reach statistical significance (Figure 1B). Analysis of 73 EAC DNA copy number profiles (GSE36460) [55] showed that the *SPP1* locus was not associated with any significant DNA copy number gain or gene amplification (Figure 1C). We confirmed the SNP results using genomic qPCR analysis in 86 pairs of matched tumor and normal esophageal samples that included the cohort of 73 EACs analyzed by SNP (Figure 1D). Thus overexpression of OPN appears to be due to transcriptional regulation. Treatment of *SPP1* endogenous low-expressing Flo cells with 5-aza-2′-deoxycytidine (decitabine), an epigenetic modifier that inhibits DNA methyltransferase activity, resulted in detectable expression of the gene, whereas abundantly *SPP1*-expressing H460 cells were not affected by treatment (Figure 1E), suggesting that *SPP1* expression could be epigenetically regulated, consistent with results recently reported in pigs [56].

### Co-overexpression of all OPN isoforms exists in primary EACs

**Figure 2:**
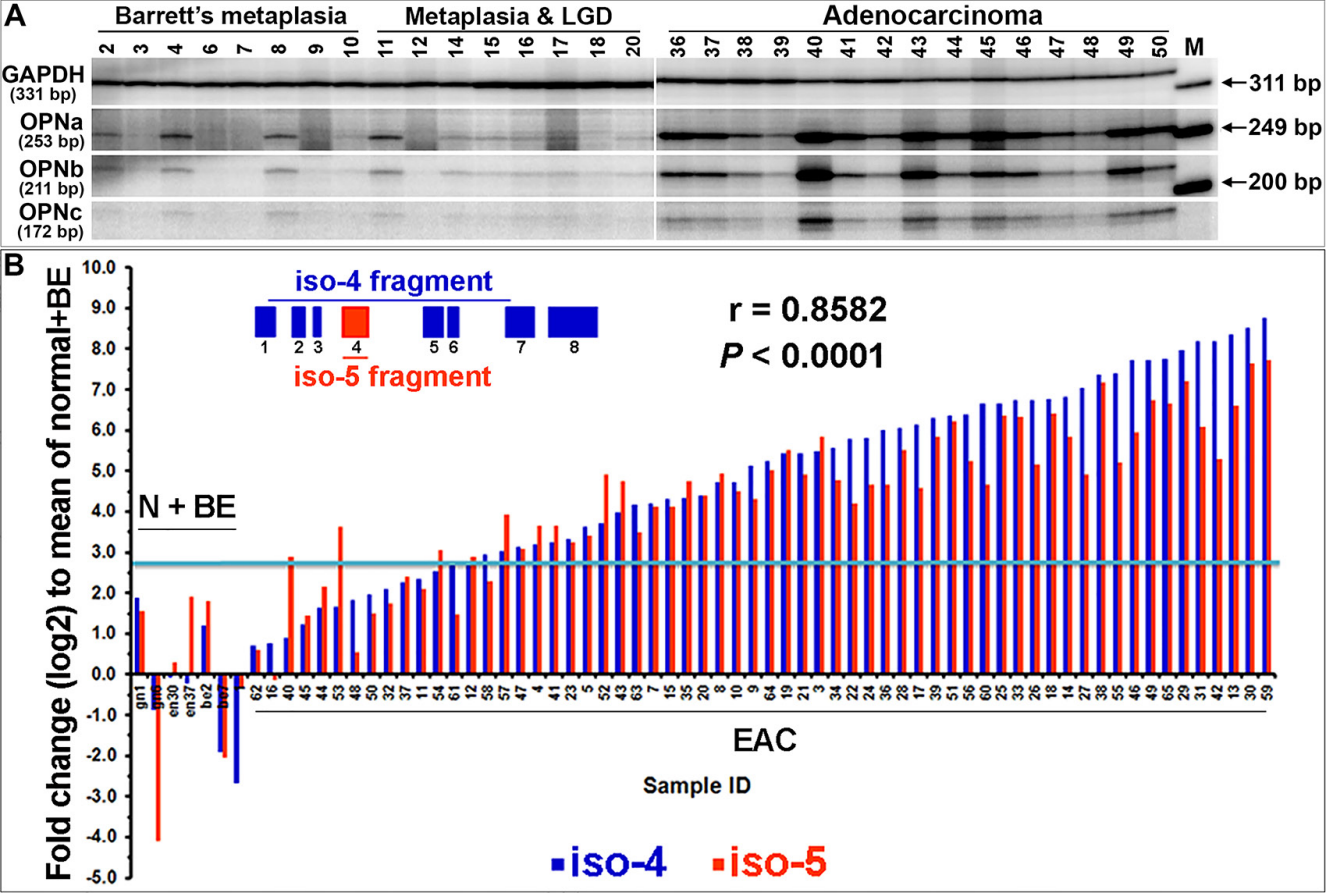
Co-overexpression of all five OPN isoforms in individual primary EACs **A.** OPN isoforms a, b and c were co-overexpressed in EACs. Quantitative RT-PCR with a pair of primers flanking the *SPP1*/OPN exons 5 and 6 and a pair of primers for *GAPDH* were co-amplified in a single-tube reaction using [γ-^32^P]-ATP labeling. Isoform fragments of OPNa (253 bp), b (211 bp) and c (172 bp), along with *GAPDH* (331 bp) were resolved by PAGE. **B.** Two sets of qRT-PCR analyses of OPN isoform 5-specific expression (primers located within exon 4) and OPN isoform 4 expression (size selection with primers crossing exons 1 to 7) were performed and a significant correlation was observed. The blue line indicates a 2-fold plus one standard deviation change as compared to the mean of normal and Barrett's metaplasia combined. (N, normal; BE, Barrett's esophagus)

**Figure 3:**
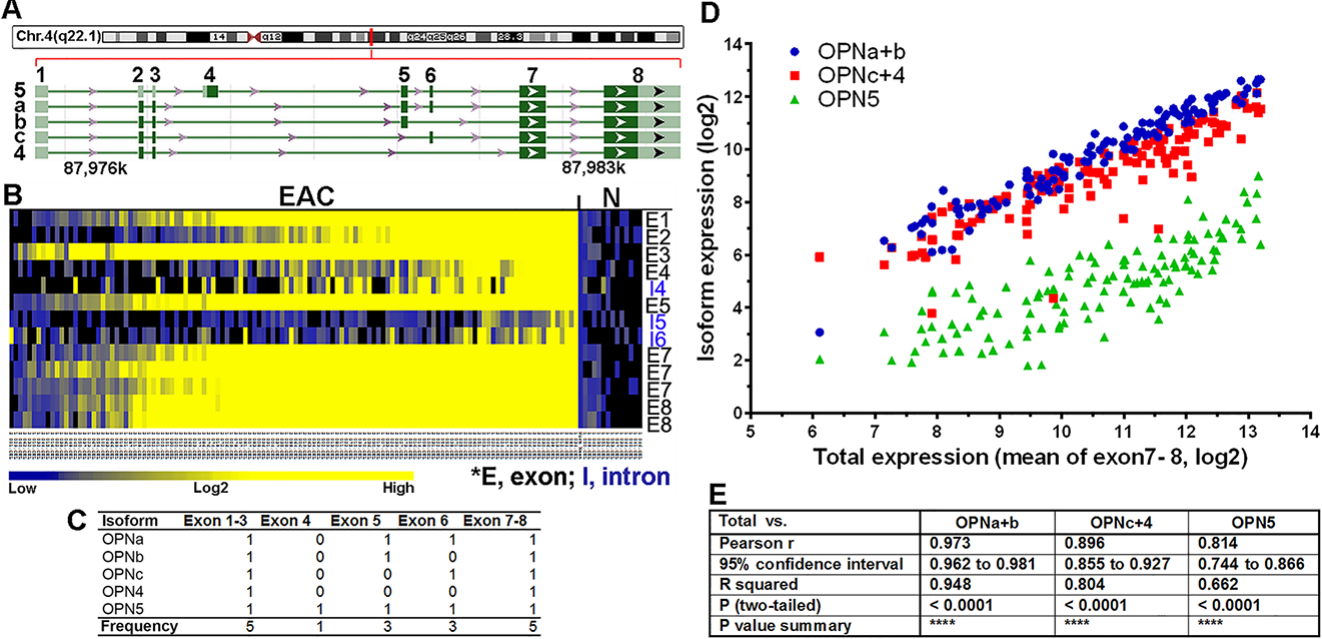
Transcriptional co-overexpression of all five OPN isoforms in primary EAC **A.** *SPP1* is located in chromosome 4q22.1, spanning 7.76 kb from 87.9756 to 87.9834 Mb in the Contig NC_000004.12. Five isoforms are banked in genome build GRCh38. OPN5 is the only isoform that has exon 4 and an alternative start codon. OPNb lacks exon 6 while OPNc lacks exon 5. OPN4 is the isoform with the shortest transcript, lacking exons 4, 5 and 6. **B.** Heat map of exome variant analysis using ST 2.1 Affymetrix arrays of 124 primary EACs (exon 6 was not available in the ST 2.1 array). **C.** Summary of OPN isoform-specific exon expression (0, absence of exon/exons; 1, presence of exon/exons; Frequency, total number of times the exon/exons is expressed across all isoforms). **D.** Correlation of OPN isoform-specific expression in primary EAC. The mean expression derived from 3 probe sets (2 from exon 7 and 1 from exon 8) with the least deviation among all common exons (1, 2, 3, 7 and 8) was used to represent the total OPN expression. Subtraction of exon 5 expression (specific for isoforms OPNa, b and 5) from the total OPN expression yielded OPNc+4 expression (red dots). Subtraction of exon 4 expression, which was specific for OPN5 (green dots), from exon 5 expression yielded OPNa+b expression (blue dots). These expression levels were then plotted against the total OPN expression for each primary EAC in the arrays. **E.** Pearson correlation coefficients showed significant correlation between these groups.

Upon further examination of *SPP1* in the NCBI database (http://www.ncbi.nlm.nih.gov/gene/6696), we noted multiple isoforms of the gene and asked whether their expression/overexpression was transcriptionally exclusive in EAC. Using specific OPN primers flanking the OPN exons 5 and 6 in single-tube [γ^32^P]ATP end-labeling RT-PCR reactions and PAGE gel analysis, we found that three isoforms, OPNa, b and c, were co-overexpressed in the majority of primary EAC samples (Figure 2A). Each OPN isoform band was gel purified and its sequence confirmed. The more recently reported OPN isoforms 4 (OPN4) and 5 (OPN5) (NCBI GRCh37) were investigated using qRT-PCR with exon 4 specific-primers for isoform 5 and primers crossing exons 1 to 7 for size-selectable qRT-PCR for isoform 4 in a cohort of 64 primary EACs (Figure 2B). We found that expression of both OPN4 and OPN5 were not only elevated in primary EACs as compared to normal and Barrett's samples but also co-overexpressed (Figure 2B). We further validated the co-overexpression of OPN isoforms using exome specific variant analysis using Affymetrix expression array ST 2.1 data for 124 primary EACs (Figure 3B). All OPN isoforms were highly overexpressed and significantly correlated (Figure 3A–3E). Exon 4 is unique to the OPN5 isoform and, therefore, showed lower relative expression compared to the other exons (Figure 3B–3D). A probe set specific for OPN exon 6, which is expressed in isoforms OPNa, OPNc and OPN5 (Figure 3A), was not available in this Affymetrix ST 2.1 array. Using the mean of three probe sets (exons 7 and 8) that represented total OPN expression and that had the smallest deviations to differentiate the specific isoforms, we were able to determine the combined isoform expression levels across EACs and to show significant correlation between the isoform groups (Figure 3D–3E). We have demonstrated that the five OPN isoforms are concurrently overexpressed in primary EACs. These data also suggest that OPN isoforms may be subject to differential transcriptional regulation, as OPNc expression was less abundant compared to OPNa and OPNb in the majority of EACs while OPNb expression was higher, lower or equal to OPNa in individual EACs (Figure 2A).

### Overexpression of OPN was confirmed using tissue microarrays (TMA)

**Figure 4:**
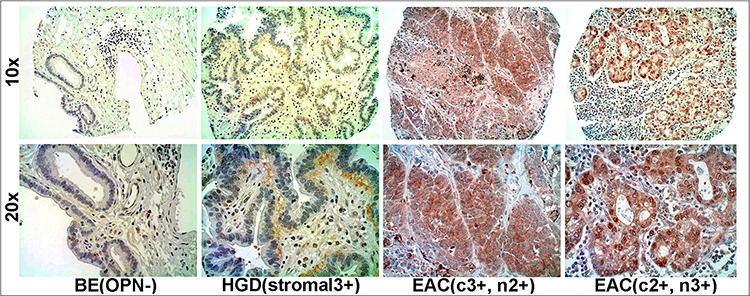
Overexpression of OPN protein in EAC using tissue microarray (TMA) immunohistochemistry Overexpression of OPN protein was observed in EACs with differential intensity but homogeneous staining. Stroma adjacent to dysplastic epithelium but not the epithelial cells themselves, overexpressed OPN (c, cytoplasmic staining; n, nuclear staining).

A TMA analyzed with an OPN antibody (Osteopontin O-17, recognizing amino acids 17–31 of exons 2 and 3 within isoforms OPNa, b, c and 4) revealed abundant cytoplasmic staining, nuclear staining or both in a subset of EAC cells that were found to have very high transcriptional expression of OPN (Figure 4). OPN protein staining tended to be homogeneously strong across positive EAC lesions. Interestingly, in premalignant esophageal dysplasia, OPN staining was predominantly localized within stromal cells beneath the dysplastic epithelia rather than in the epithelial cells themselves (Figure 4).

### Ectopic expression of individual OPN isoforms in EAC cells lacking endogenous OPN expression

Having confirmed transcriptional co-expression of individual OPN isoforms in primary EACs, we went on to determine the functional similarities and differences of OPN isoforms in relation to tumor progression. We individually cloned each of three isoforms, OPNa, OPNb and OPNc, into expression constructs using the OPN highly-expressing H460 (large cell lung carcinoma) cell line as the template (Figure 3A, Figure S2A–S2C). The EAC cell lines, Flo, OE33 and OE19, have low base-line endogenous levels of OPN (Figure S2A). Both Flo and OE33 cells were stably-transfected with each individual isoform (Figure S2B). Pooled transfectants of each isoform were cultured for > 10 passages to ensure adaptation and consistent expression across experiments (Figure S2C–S2E). In addition, stable transfectants cloned at the thrombin cleavage site (R/SK) for the three isoforms were also established in Flo and OE33 cells, designated as OPNia, OPNib and OPNic lines (Figure S2B). These latter three transfectants include the integrin domains (RGD and SVVYGLR) but not the C-terminal CD44 domain.

### OPNb and OPNc act distinctly in cancer cell invasion and migration

**Figure 5:**
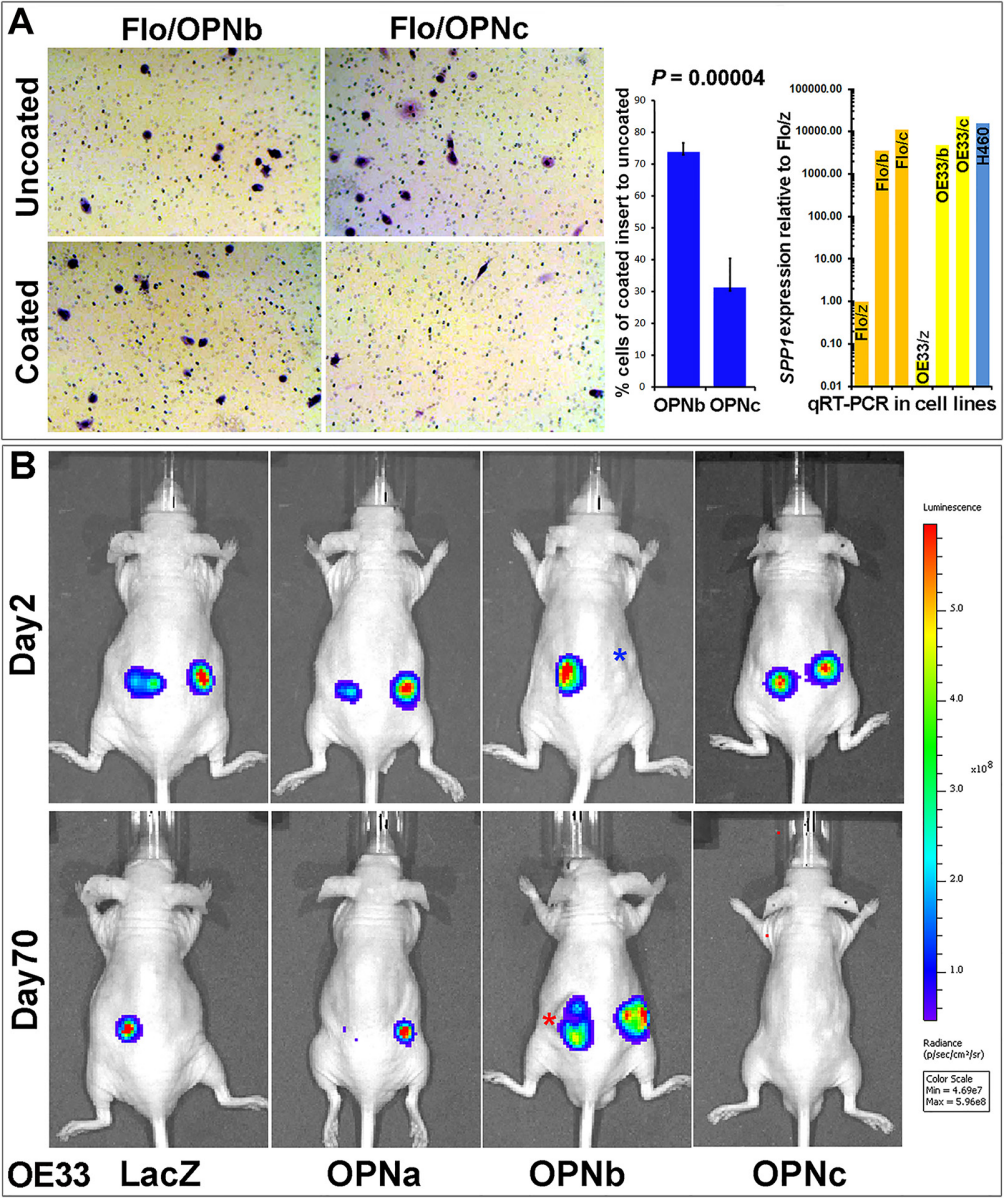
OPNb- and OPNc-expressing stable cells differ in cancer cell invasion **A.** OPNb cells showed significantly more invasion than OPNc cells using Matrigel Basement Membrane Matrix-casted culture dishes (250 μg/ml of BD Matrigel Matrix) following 24–36 h incubation and Diff-Quick staining as compared with cells in non-casted culture dishes. OPN isoform expression levels were monitored using qRT-PCR. **B.** Matrigel Matrix-resuspended OPNb cells displayed more invasive growth and xenograft formation *in vivo* than OPNc cells. One million Lenti-Luc-labeled OE33/OPN stable cells were resuspended in 0.1 ml Matrigel Matrix and subcutaneously injected into the flanks of nude mice. *In vivo* tumor imaging to monitor growth was performed using a Xenogen IVIS Spectrum scanner (*Note, red asterisk, actual imaging intensity should be greater than the reported measurement (total flux, p/s) due to tumor ulceration; blue asterisk, nodule at the site of subcutaneous injection but luciferin signal not detected).

We observed that isoforms OPNb and OPNc contributed to a variety of distinctive phenotypes in cultured EAC cells as well as in EAC cell xenografts grown in nude mice. OPNb stable cells demonstrated significantly enhanced cell invasion *in vitro* as compared to OPNc cells in Matrigel assays (Figure 5A). When OE33/OPN isoform stable cells were labeled with luciferase, resuspended in Matrigel solution and injected into the flanks of nude mice, OPNb cells invaded Matrigel and grew into significantly larger xenografts in mice while OPNc cells failed to produce any tumor xenografts (Figure 5B). Expression levels of individual OPN isoforms were monitored in each experiment using qRT-PCR as represented in Figure 5A and Figure S2D–S2E.

**Figure 6:**
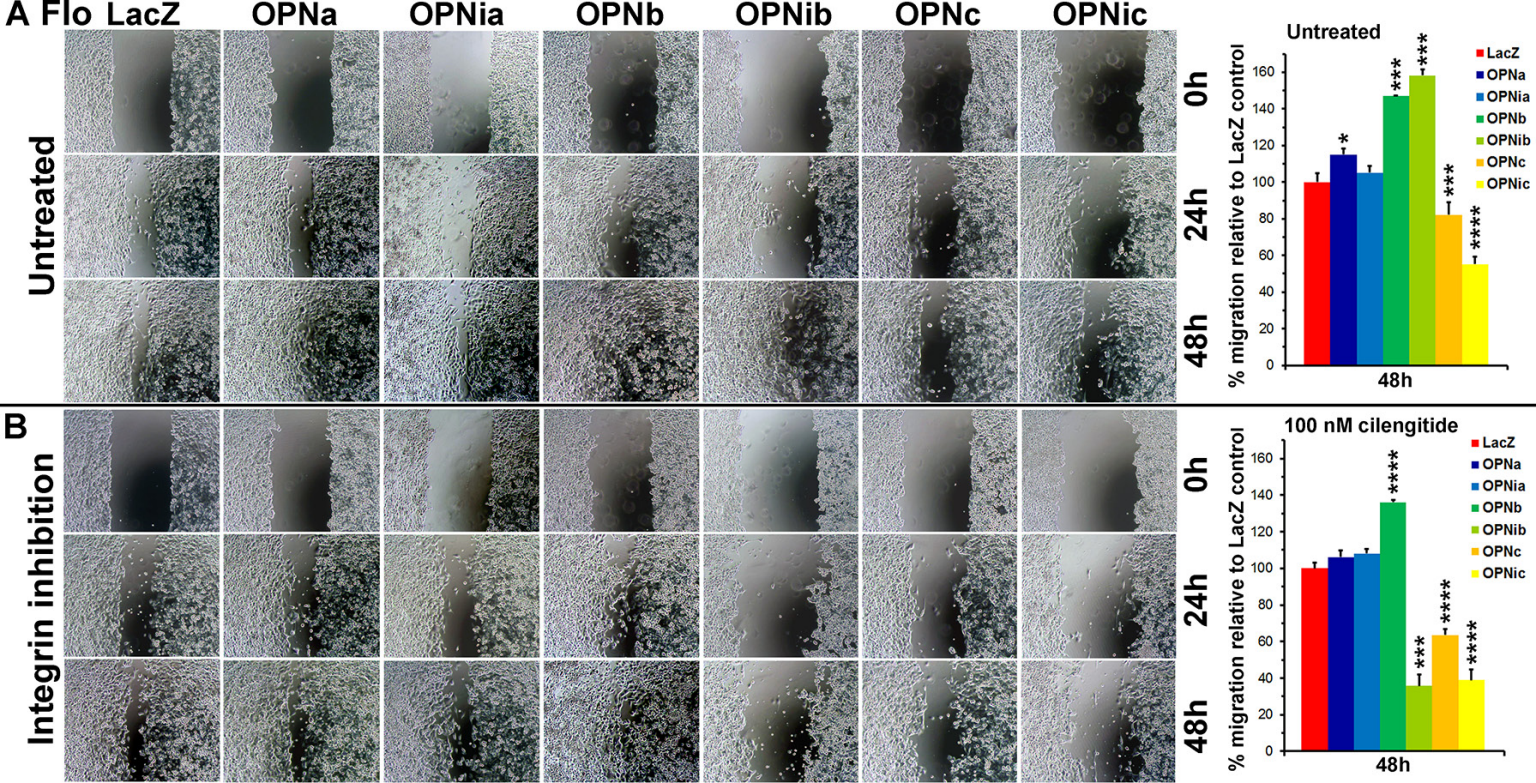
OPN isoform-expressing stable cells differ in cell migration and their response to RGD integrin inhibition Flo/OPN stable cells were seeded at 0.6 × 10^6^ cells/12-well, wounded and cultured in media in the presence or absence of cilengitide (100 nM) up to 96 h post wounding. **A.** In the absence of cilengitide, both OPNb and OPNib cells showed significantly increased migration as compared to LacZ control cells. Both OPNc and OPNic cells were significantly less motile, with gaps remaining more than 96 h post wounding. OPNa cells showed increased migration as compared to LacZ control cells whereas OPNia cells demonstrated similar migration to LacZ cells. **B.** In the presence of cilengitide, OPNb cell motility was attenuated while OPNib cell migration was severely hindered. Reduced migration of OPNc and OPNic cells remained unchanged. Increased motility of OPNa cells was also attenuated but OPNia cell migration was similar to LacZ cells (*P < 0.05, ***P < 0.001, ****P < 0.0001, *t*-test).

OPN has been known to bind and activate both integrin and CD44 [28, 29, 33]. Both integrin and CD44 receptors are involved in cell adhesion, in which they lead to transduction of overlapping signaling pathways that mutually interact as an OPN-integrin-CD44 axis in tumor progression [15, 35, 37, 38]. To elucidate the specific functionality of each OPN isoform, we built not only full-length OPN isoforms a, b and c expression vectors but also corresponding expression constructs lacking the CD44 domain, designated as OPNia, OPNib and OPNic (for integrin domain only isoforms OPNa, b and c), which are endogenously produced following thrombin cleavage of full length isoforms at the RSK site. We found that OPNb cells took significantly less time to migrate into and to close a wound as compared to LacZ cells using the wound-healing assay (Figure 6A). Although slower than OPNb cells, OPNib cells similarly migrated much faster than LacZ cells, suggesting that the presence of the integrin domain of isoform OPNb is sufficient to sustain cell migration (Figure 6A). Moreover, migration of OPNb cells was attenuated when OPN antibody was present in the culture media (Figure S3A). Conversely, both OPNc and OPNic required extensively longer times or did not close the wound as compared to LacZ control cells (Figure 6A). OPNa cells closed the wound in a significantly shorter time than LacZ cells but migration of OPNia cells did not differ from LacZ control cells (Figure 6A). When OPN-null OE19 EAC cells were cultured with isoform-specific donor-media from individual Flo/OPN stable cells, OPNb-media promoted significantly more cell migration than OPNc-media (Figure S3B) consistent with the results in OPN isoform-expressing stable cells (Figure 6A). Cilengitide is a cyclicized RGD pentapeptide that selectively and potently inhibits the major OPN integrin receptors αVβ3 and αVβ5. When wounded OPN stable cell cultures were treated with 100 nM cilengitide, cell motility of OPNb cells was attenuated whereas the migration of OPNib cells was severely hindered (Figure 6B). The decreased cell mobility of OPNc and OPNic cells did not change following cilengitide treatment (Figure 6B). While mobility of OPNia cells also remained unchanged by integrin inhibition, migration of OPNa cells was reduced as compared to treated LacZ cells and untreated OPNa cells (Figure 6B). These results suggest that OPNb-enhanced cell motility is strongly dependent on integrin binding as evidenced by both the significant increase in and subsequent cilengitide-effected abrogation of cell migration in OPNib cells. These data also suggest that OPNa isoform-induced cell motility is weaker than OPNb-mediated cell migration and that CD44 is necessary along with integrin signaling to sustain this mobility, supported by the similar migration of OPNia and LacZ control cells and reduced OPNa cell migration upon integrin inhibition. Stable expression of OPNc significantly reduced cell motility and these cells did not respond to integrin inhibition.

### OPNb cells exhibit significantly increased cell adhesion while OPNc cells show enhanced cell detachment

**Figure 7:**
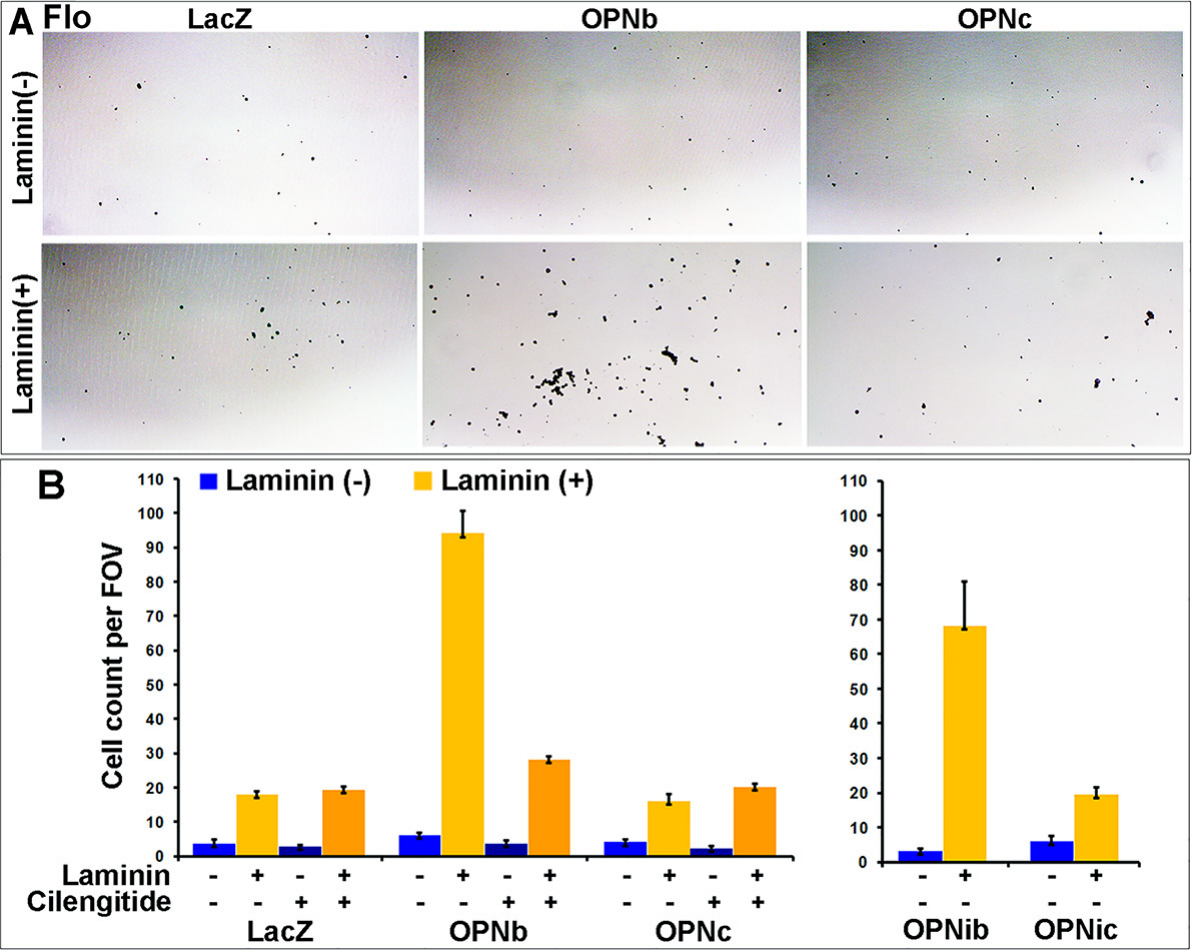
OPNb- and OPNc-expressing cells differ in cell adhesion and their response to RGD integrin inhibition Culture plates were coated with Matrigel mixture (laminin at 10.8 μg/ml), blocked for 30 min, seeded with a single cell suspension (0.01 × 10^6^ cells/96-well) and incubated at 37°C/5% CO_2_ for 30 min. Cells were then fixed with glutaraldehyde and stained with Diff/Quick solution. **A.** Representative images demonstrating that OPNb significantly enhanced cell adhesion in laminin casted culture plates as compared to the LacZ control and OPNc stable cells. **B.** Enhanced cell adhesion was also observed for OPNib- but not OPNic-expressing cells. The significant increase in OPNb cell adhesion was abrogated with the addition of 1000 nM cilengitide. (FOV, field of view)

**Figure 8:**
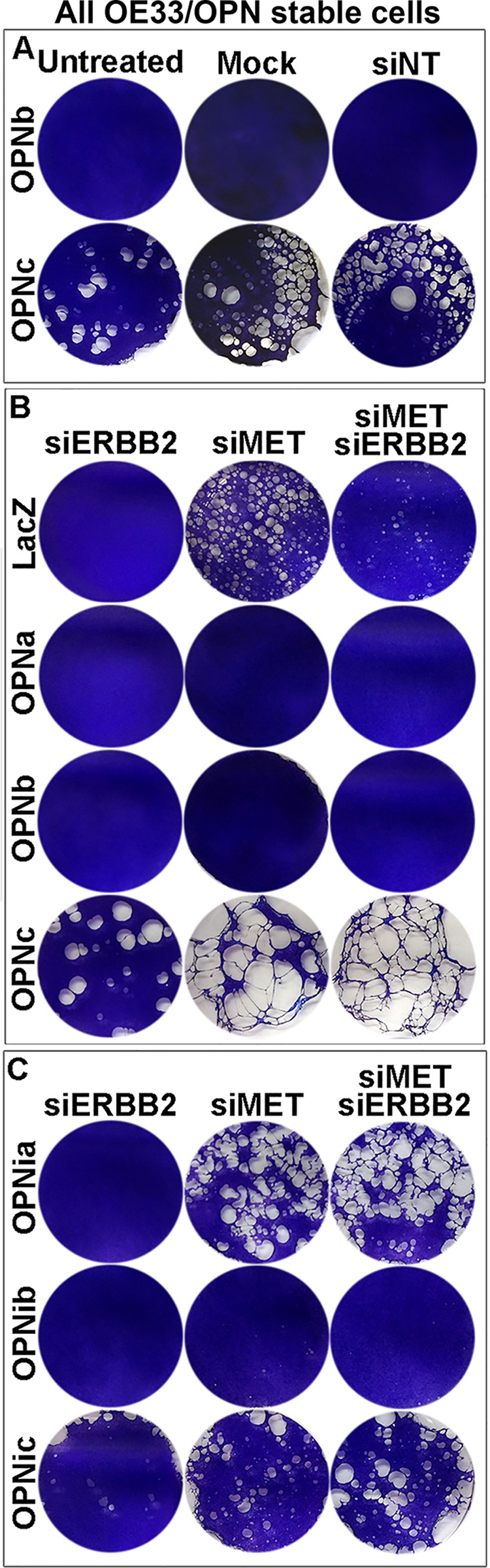
OPN isoform-expressing cells differ in cell detachment phenotypes that can be modulated by the *MET* oncogene **A.** Significant cell detachment was observed for OE33/OPNc-expressing cells. OE33/OPN stable cells were seeded in culture dishes at 80% confluence (0.22 × 10^6^ cells/ml in 6-well plates) for 24 h, transfected with lipofectamine alone (mock) or siNonTarget (siNT) or untreated for 96 h and then fixed and stained with Diff-Quik staining solution. While OPNb-expressing cells demonstrated adherence to culture dishes, OPNc cells were significantly detached, as exhibited by markedly increased non-stained areas in culture dishes (see also Figure S4A). **B.** OE33 cells harbor both *MET* and *ERBB2* gene amplification with corresponding gene overexpression (see Figure S4C). Silencing of MET using siRNA enhanced OE33/OPNc cell detachment while knockdown of *ERBB2* did not alter its existing phenotype. Expression of both OPNa and b in OE33 cells appeared to increase adhesion of these cells, as control LacZ cells exhibited increased detachment following transfection with 10 nM *siMET* or co-transfection with 5 nM each *siMET* and *siERBB2* as compared to transfection with 10 nM *siERBB2* alone. **C.** Silencing of MET resulted in significant detachment of OPNia-expressing cells but did not change the phenotypes of OPNib or OPNic-expressing cells as compared to their full-length OPN counterparts treated with *siMET*.

We observed that OE33/OPNc cells required shorter trypsinization time than OPNb cells and that they significantly detached in culture when grown to semi-confluence as compared to LacZ and OPNb stable cells (Figure S4A). We found that these detached cells were able to grow in culture after collection and replating. This distinct OPNc phenotype may be explained by what we found in OE19 cells that were chronically treated with OPNc-media (Figure S4B). When diluted OPN-null OE19 cells were chronically cultured with OPN isoform-specific donor-media collected and filtered from Flo/LacZ, OPNa, OPNb and OPNc stable cells, most OE19 cells treated with LacZ, OPNa and OPNb donor-media grew as single-layered foci whereas most OE19 cells cultured with Flo/OPNc-media exhibited multi-layered spherical-shaped foci (Figure S4B). We further assessed cell adhesion in OPNb and OPNc cells using adhesion assays and found that OPNb cells were significantly more adherent to laminin-coated culture dishes than OPNc and LacZ cells (Figure 7A). Treatment of OPNb cells with the integrin inhibitor cilengitide abrogated this increased adhesion (Figure 7B). OPNib cells also showed enhanced cell adhesion, indicating that OPNb-mediated integrin signaling alone supports increased cell adhesion (Figure 7B). Given that significant cell detachment was detected in OE33/OPNc (Figure S4A) but not Flo/OPNc stable cells, that gene amplification and amplification-related overexpression of both *ERBB2* and *MET* occur in OE33 but not in Flo cells (Figure S4C) and that previous studies have demonstrated activation of *MET* by CD44v6 and integrins [57–61] and an invasive growth program resulting from *MET* signaling [62–64], we examined whether these amplifications affected the cell adhesion phenotype in OE33/OPNc cells. We knocked down *ERBB2* alone, *MET* alone or both together using siRNAs against these two genes in OE33/OPN cells. Treated cells were allowed to grow to confluence and then fixed and stained with the Diff-Quik Staining System (Figure 8). We observed that knockdown of *ERBB2* did not change the phenotype; however, silencing of MET significantly enhanced cell detachment, which was evidenced by reproducibly-increased unstained areas in culture dishes containing OPNc cells treated with *siMET* as compared to untreated, mock-treated, non-target siRNA-treated, or *siERBB2* treated cells (Figure 8A–8B). These results are consistent with the role of MET in invasive growth in that silencing of MET augments OPNc-induced cell detachment. In addition, we found that OE33/LacZ but not OPNa or OPNb cells exhibited increased detachment upon the knockdown of *MET*, indicating that OPNa and OPNb enhanced cell adhesion to counteract cell detachment induced by the knockdown of *MET* (Figure 8B). Interestingly, OPNia stable cells were more detached than OPNa cells following treatment with *siMET* or *siMET*+*siERBB2* whereas detachment in OPNic cells upon silencing of MET was more similar to the detachment seen in OPNc cells without *siMET* treatment (Figure 8A–8C). OPNib cells retained their adhesive properties when transfected with *siMET* (Figure 8C). The expression and efficiency of siRNA knockdown of *ERBB2* and *MET* were monitored by qRT-PCR. Cell adhesion/detachment assays were repeated and results were confirmed in three independent experiments.

These results suggest that OPNb strongly enhances cell adhesion and attachment in the presence or absence of CD44 signaling and that OPNb-integrin signaling alone is sufficient to withstand *siMET*-induced cell detachment. While OPNa sustains cell adhesion and attachment, the presence of the CD44 domain is required to antagonize *siMET*-induced cell detachment. While OPNc promotes cell detachment with or without CD44 signaling, the *siMET*-enhanced detachment of OPNc cells is likely CD44-dependent.

### Inhibition of RGD integrins significantly reduces 2-D clonal growth of OPNb stable cells only and does not change 3-D colony formation of EAC/OPNb or OPNc cells

**Figure 9:**
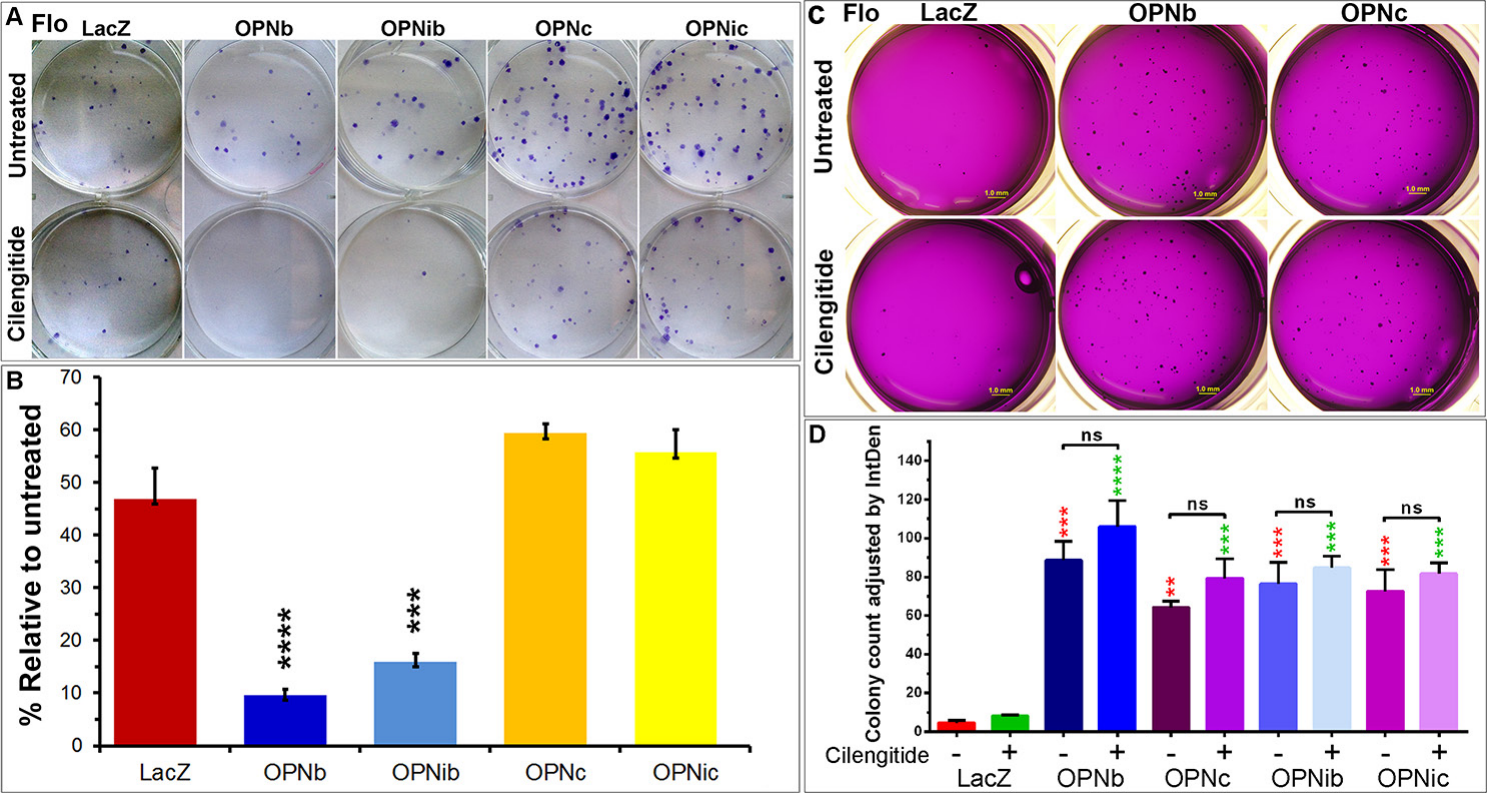
Integrin inhibition differentially influences cell growth in 2-D and 3-D cultures of OPNb- and OPNc-expressing cells **A.** Cilengitide reduces OPNb but not OPNc cell proliferation in 2-D cultures. Representative plates were shown. OPNb, OPNib, OPNc, OPNic stable and LacZ control cells were seeded in sextuplicate at 150 cells/6-well. Sets of 3 wells were mock-treated or treated with IC_50LacZ_ dose of cilengitide (1000 nM) for 12 days, and clonal foci were fixed and stained with Diff-Quik Staining solution. **B.** Bar graph representation of stained foci showing significantly reduced OPNb and OPNib cell proliferation in the presence of cilengitide as compared to their untreated cells, which exceeded the 50% (IC_50LacZ_) reduction observed in treated LacZ control, OPNc or OPNic cells. (***P < 0.001, ****P < 0.0001, *t*-test) **C.** Ectopic expression of both OPNb and OPNc enhanced EAC cell colony formation in 3-D soft agar cultures. Integrin inhibition did not significantly alter the colony formation of these cells. Flo/OPN and LacZ control cells (7000 cells/12-well, from the same cell-suspensions in Figure 9A) were mixed with top-agar media in the presence or absence of cilengitide (1000 nM) and were pipetted onto the casted base-agar with or without cilengitide. Cells were allowed to grow for 20 days with periodic replacement of the overlaid media with or without cilengitide. Agar plates were stained with 0.05% crystal violet in 50% ethanol and imaged using the Leica MXFL III Stereo microscope; Olympus DP-70 digital camera at × 0.8 magnification. Soft agar assays were performed in triplicate. **D.** Significantly increased colony-formation was observed in Flo/OPNb, OPNib, OPNc and OPNic cells as compared to Flo/LacZ controls and was not altered with the addition of cilengitide. Colonies were counted using ImageJ software, and graphs were generated using Prism software. (red, comparison to untreated LacZ; green, comparison to cilengitide-treated LacZ; ns, not significant; **P < 0.01, ***P < 0.001, ****P < 0.0001, One-way ANOVA)

An IC_50(LacZ)_ dose of cilengitide significantly reduced the clonogenic survival of Flo/OPNb and OPNib cells but not OPNc or OPNic cells in regular 2-D culture dishes as compared to LacZ cells in clonogenic assays (Figure 9A–9B). Both Flo/OPNb and OPNc cells demonstrated significantly increased colony formation as compared to Flo/LacZ control cells in soft agar assays (Figure 9C). Interestingly, this enhanced colony formation was not affected in both OPNb and OPNc cells when cultured in 3-D soft-agar in the presence or absence of 1000 nM cilengitide (Figure 9C–9D).

These data further indicate that both OPNb and OPNc enhance cellular anchorage-independent growth of transformed cells and that anchorage-independent growth is not affected by integrin inhibition. Conversely, we show that OPNb-mediated cell proliferation is integrin-dependent while OPNc most likely acts via an integrin-independent pathway.

### Differential expression of E-cadherin and vimentin is observed in individual OPN isoform stable cells

**Figure 10:**
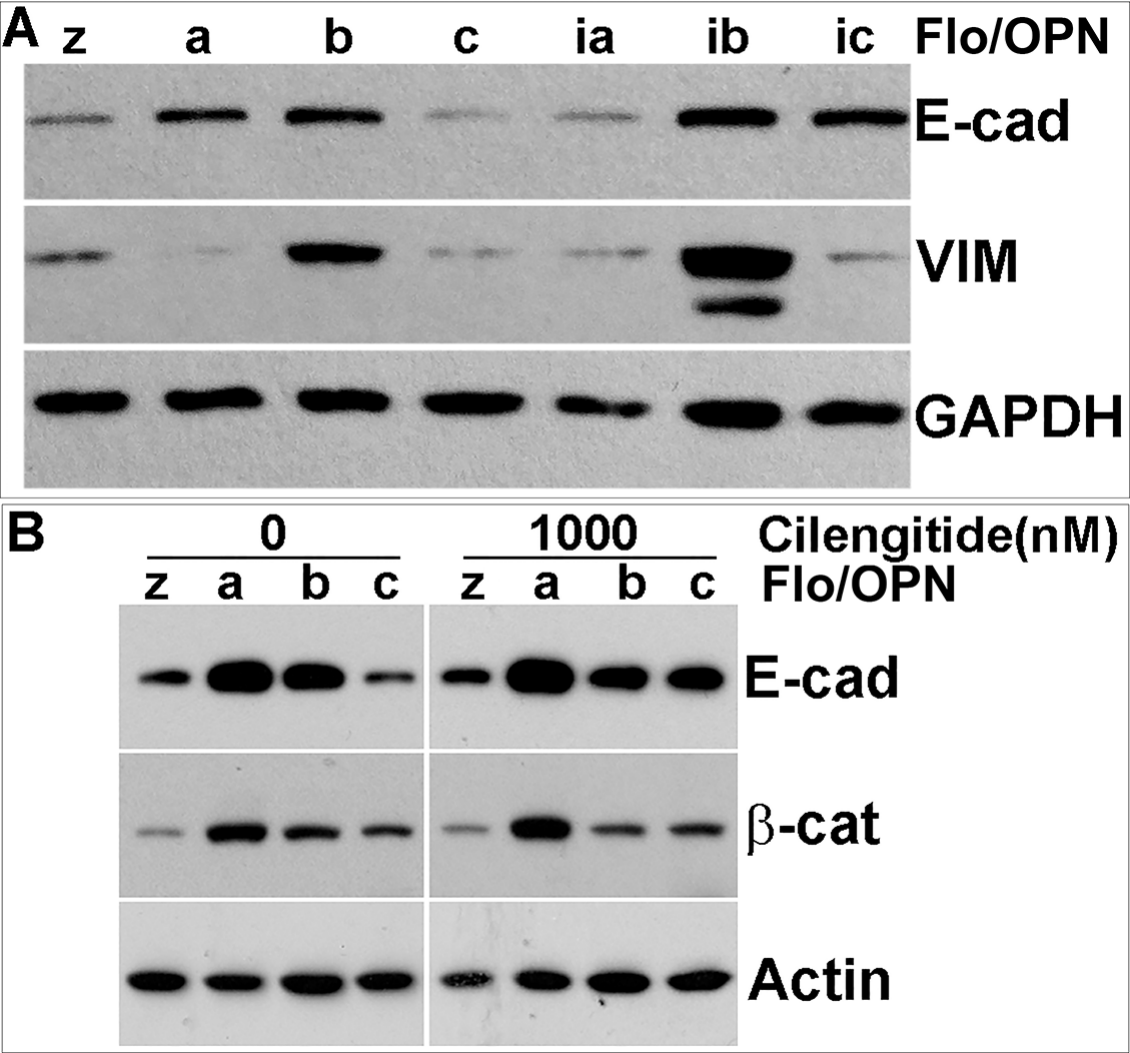
OPN isoform stable cells express different levels of WNT signaling- and EMT-related proteins **A.** Western blot analysis revealed increased E-cadherin expression in OPNa-, OPNb-, OPNib- and OPNic-expressing cells but decreased expression in OPNc-and OPNia-expressing cells as compared to LacZ controls. Vimentin expression was increased in both OPNb- and OPNib-expressing cells but decreased in OPNa-, OPNia-, OPNc-, and OPNic-expressing cells. **B.** Elevated β-catenin expression was observed in OPNa-, OPNb- and OPNc-expressing cells. Addition of cilengitide did not alter β-catenin expression in any of the OPN isoform-expressing cells but induced E-cadherin expression in OPNc-expressing cells. GAPDH or β-actin was included as a loading control.

Having observed altered adhesion and detachment phenotypes in OPN isoform-expressing stable cells, we wanted to know whether ectopic stable expression of OPN isoforms altered expression of other classes of adhesion molecules. We found that while E-cadherin expression was elevated in OPNa, OPNb, OPNib and OPNic stable cells as compared to LacZ cells, it was decreased in OPNc and OPNia cells (Figure 10A–10B). Expression of the mesenchymal marker vimentin was markedly increased in OPNb and OPNib cells as compared to other isoform stable and LacZ control cells (Figure 10A). Increased β-catenin expression was found in OPNa, b and c relative to LacZ cells, with the lowest increase found in OPNc cells. In the presence of cilengitide, this upregulation of β-catenin as well as E-cadherin was slightly decreased in OPNb cells but unchanged in OPNa cells. Additionally, cilengitide treatment elevated the expression of E-cadherin and β-catenin in OPNc cells (Figure 10B). The differential expression of E-cadherin, β-catenin and vimentin in individual OPN isoform stable cells could contribute to the morphologic and functional differences observed in the OPN isoform-expressing stable cells.

## DISCUSSION

Understanding the molecular basis underlying early invasion and metastasis of EAC may lead to new and more effective therapeutic interventions resulting in improvement in the prognosis of patients with EAC. Osteopontin (OPN) has been reported as a cytokine-mediated cell adhesion and motility factor that likely acts via both CD44 and integrin receptors [29, 65]. Several studies have suggested that OPN potentiates site-specific metastasis and that secreted OPN, derived from aggressive cancer cell xenografts, instigates indolent tumor cells to actively grow *in vivo* [15, 25, 66, 67]. Recently, it has been suggested that OPN may play a key role in maintaining stemness-like phenotypes in hepatocellular carcinoma (HCC) cell lines via αvβ3/NFκB/HIF1α signaling [68]. We demonstrated here that OPN is highly and frequently overexpressed in primary EACs. Using conventional end-labeling quantitative RT-PCR and bioinformatic exome variant analysis of the Affymetrix array ST 2.1 in a large cohort of EACs, we showed that all five OPN isoforms are co-overexpressed in primary tumors. Systematic comparison analyses of EAC OPNb- and OPNc-transfected stable cells demonstrated distinct phenotypes in cell adhesion, migration, *in vitro* and *in vivo* invasion, cell detachment and proliferation. Our data also showed that while OPNb and OPNa functioned through integrin signaling, OPNc acted in an integrin-independent manner. These functional differences could be partially accounted for by variable expression of E-cadherin, β-catenin and vimentin in stable OPNa, OPNb and OPNc cells, respectively. Silencing of MET in *MET*-amplified OE33/OPNc stable cells augmented OPNc-induced cell detachment, yet OPNb-enhanced cell adhesion was not affected. While expression of both OPNb and OPNc in the transformed EAC cells significantly boosted the number of colonies formed in soft agar, blockade of integrin signaling did not affect their anchorage-independent growth. RGD integrin inhibition did, however, significantly reduce OPNb stable cell proliferation in 2-D cultures.

The finding that OPN is highly and frequently overexpressed in a large cohort of primary EACs at both the transcriptional and protein levels as reported here supports data from a previously reported expression profiling study of EAC and a multi-tumor analysis that included 10 cases of esophageal squamous carcinoma, 16 cases of pancreatic duct adenocarcinoma and 11 cases of gastric adenocarcinoma with OPN up-regulated in 70%, 81% and 100% of cases, respectively [48, 69]. Increased expression of OPN has also been identified in many other types of cancer and reported as either a biomarker or a functional regulator of cancer invasion and metastasis [28, 39, 70–73]. Since its identification in the late 1970′s, OPN has been studied extensively as a cytokine and as a non-collagenous component of the extracellular matrix [29, 33, 74, 75]. In the present study, we observed intense OPN staining predominantly in the primary EAC cells that had high RNA overexpression and only rare staining in the surrounding stromal matrix. In contrast, stromal cells adjacent to dysplastic epithelial cells showed much stronger OPN staining than the epithelial cells themselves. The data suggest a switch in expression from the surrounding stroma to tumor cells during transformation from premalignant high grade dysplasia (HGD) to EAC. Copy number alterations were not related to *SPP1*/OPN overexpression. However, epigenetic mechanisms might influence OPN expression levels, as the hypomethylating agent decitabine induced OPN expression in endogenously low- or non-expressing EAC cells. Epigenetic modification of OPN has been previously reported using trichostatin A (TSA), a potent histone deacetylase (HDAC) inhibitor [76, 77]. OPN has also been shown to be regulated by v-Src, PDGF, middle T antigen, and mechanically-induced reduction of methylation in the OPN promoter region [75–80]. Hormones, cytokines, and several other growth factors have been suggested to regulate OPN as well [33].

Five OPN isoforms have been reported in the GenBank database (http://www.ncbi.nlm.nih.gov/genbank/). With five transcriptionally spliced OPN isoforms, multiple post-translational modifications (phosphorylation, glycosylation and sulfation) and multiple functionally-active proteolytic forms cleaved by thrombin, MMPs, plasmin and cathepsin D, OPN is a versatile modulator of many physiological and pathological processes, including cancer progression [33, 81–87]. In this study, we attempted to address the question of whether these spliced isoforms are transcriptionally exclusive or inclusive in individual tumors, as previous evidence has been inconclusive [88]. We demonstrate that the five OPN isoforms, OPNa, OPNb, OPNc, isoform 4 and isoform 5, are co-upregulated in primary EACs. A previous study in hepatocellular cancer reported that OPNc was predominantly expressed in non-malignant tissues and was associated with non-migratory phenotypes [89]. Our data suggest that OPNc not only reduces cell motility and enhances cell detachment but is also present in primary tumors. Our data also partially differ from a previous report in ovarian cancer that OPNc is the only tumor-specific isoform [90]. With all OPN isoforms co-overexpressed in EAC, the present study suggests that each OPN isoform has a distinct phenotype yet acts collectively to promote EAC progression. OPNb stable cells displayed enhanced cell proliferation, enhanced cell migration and invasion, yet sustained more cell adhesion. OPNc stable cells showed decreased cell migration and invasion but enhanced cell detachment. Stable expression of both OPNb and OPNc in EAC cells enhanced anchorage-independent growth in 3-D soft agar cultures. Some of these findings are consistent with previous observations in liver and breast cancers [52, 89]. Although less than its full-length counterpart, motility of OPNb cells lacking the CD44 domain was still significantly greater than LacZ cells and other OPN isoform cells. Integrin inhibition in Flo/OPN isoform stable cells attenuated migration of OPNb cells but completely halted the migration of OPNb cells lacking the CD44 domain, strongly suggesting that OPNb is highly integrin-dependent. The finding that OPNa and OPNb are integrin signaling-dependent while OPNc is integrin-independent has not been previously reported.

Interestingly, increased co-expression of E-cadherin and vimentin was observed in both OPNb and OPNib cells. While OPNa cells showed significantly increased E-cadherin and β-catenin, OPNc cells expressed minimal vimentin. Decreased E-cadherin and increased vimentin are indicative of epithelial-mesenchymal transition (EMT) in cancer, and elevated E-cadherin suppresses tumor invasion [91]. However, recent studies have reported that frequent, high level expression of E-cadherin is observed in colonized metastatic-cancer cells [92, 93] and that high expression of E-cadherin is associated with aggressive tumor growth [94]. Co-expression of epithelial proteins, such as E-cadherin, and mesenchymal markers, such as vimentin, has been observed in the majority of circulating tumor cells (CTC) of metastatic prostate and breast cancer patients [95].

In summary, we demonstrated that all five isoforms of the single gene, *SPP1*, are co-expressed in the majority of primary EACs and act collectively in promoting tumor cell invasion and dissemination. Because all isoforms are co-expressed in primary tumors and function differently in the metastatic cascade, it is necessary to strategize a therapeutic targeting regimen aimed at both integrin-dependent and -independent OPN signaling cascades. Further investigation will be required to better characterize these critical isoform-signaling pathways in EAC.

## MATERIALS AND METHODS

### Patients and EAC samples

Written consent was obtained from all patients and the protocols received approval from the Institutional Review Board (IRB) at the University of Michigan. Tissues were obtained from patients undergoing esophagectomy for adenocarcinoma at the University of Michigan Health System between 1991 and 2011. Patients in this study had no preoperative radiation or chemotherapy unless otherwise noted. Specimens were fresh-frozen in liquid nitrogen and stored at −80°C until use. Cellularity of metaplastic, dysplastic and tumor samples were determined by cryostat sectioning to be greater than 70% before DNA, RNA, or protein isolation. High molecular weight DNA was isolated as described previously with modifications [96]. RNA was isolated using the QIAGEN RNeasy Mini Kit and treated with DNase I following the manufacturer's instructions (QIAGEN). All DNA or RNA was quantified using the NanoDrop 3000 (NanoDrop Products). All RNA samples were subjected to gel electrophoresis or Bioanalyzer analysis for integrity assessment. Protein was isolated using lysis buffer (Cell Signaling Technology) with the addition of fresh protease inhibitor cocktail (Sigma-Aldrich). Lysates were centrifuged at 14, 000 rpm, 4°C for 20 minutes (min), and the supernatant was collected and stored at −80°C. Protein was quantified using the *DC* Protein Assay Kit II (Bio-Rad) following the manufacturer's instruction and absorbance was read at 750 nm using an FLx800 Fluorescence Microplate Reader (BioTek, Winooski, VT).

### Affymetrix microarray assays

Esophageal specimens, including nondysplastic and dysplastic Barrett's mucosa and EACs were subjected to gene expression profiling using Affymetrix U133A or ST 2.1 chips (Affymetrix, Santa Clara, CA) as previously described [97, 98]. Gene expression profiling data were deposited to the Gene Expression Omnibus (GEO) database (accession numbers: GSE37200 and GSE37201). A heat-map was computed and generated using Cluster and TreeView software (http://rana.lbl.gov/EisenSoftware.htm).

### Real-time RT-PCR (qRT-PCR) and [γ-^32^P] -labeled quantitative RT-PCR analyses

All PCR and RT-PCR primers were designed using the DNASTAR PrimerSelect software (DNASTAR, Inc. Madison, WI) and tested for specificity by gel electrophoresis (representative image in Figure S1A). Individual annealing temperatures were optimized using the Cepheid Smart Cycler (Cepheid). The melt curves of all qPCR or qRT-PCR reaction were closely monitored to avoid non-specific SYBR green signals (Figure S1B; Figure S2D–S2E). Copy number or gene fold changes were determined by the 2^−ΔΔCt^ algorithm [99]. Duplicate reactions were performed by two or three individual researchers and the correlation analyzed (Figure S1B–S1C). An index of 3 reference genes (*GAPDH*, *ACTB*, and *RPLP0*) [100] was applied to normalize the 2^−ΔΔCt^ -based assessments. All primer sequences used are listed in Table S1 or previously published [100]. Details for [γ-^32^P]-ATP (NEN Life Science Products, Boston, MA) end-labeling for qRT-PCR analyses were as previously described [101]. The ΦX174 DNA/HinfI ladder was used as a reference (Promega).

### Immunohistochemistry of tissue microarrays (TMA)

TMAs were constructed as previously described [102, 103]. Briefly, TMA arrays contained 122 cores from formalin-fixed paraffin-embedded tissue blocks from 73 EAC patients, including 63 EAC, 18 mixed EAC and dysplasia, 22 Barrett's metaplasia and dysplasia, 9 lymph node metastases and 10 from various types of normal tissue. TMA slides were hybridized with polyclonal OPN antibody (Cat. # 915-034, clone O-17, Assay Designs Inc, Ann Arbor, MI) at a 1:750 dilution, following microwave citric acid epitope retrieval. Slides were lightly counterstained with hematoxylin. Each sample was then scored 0, 1, 2, or 3 corresponding to absent, light, moderate, or intense staining by two individual researchers.

### Cell lines, culture conditions, cloning strategies and gene expression constructs

The Flo cell line (derived from a stage IIB EAC patient in our laboratory) as well as both OE33 and OE19 cell lines (Sigma-Aldrich) were cultured in DMEM and RPMI media, respectively, supplemented with 10% FBS and 1% antibiotic-antimycotic agent (GIBCO) unless otherwise noted. All cell lines and stable subclones were subjected to genotyping by the University of Michigan Sequencing Core facility to ensure cell line authenticity. Because endogenous expression of OPN in Flo, OE19 and OE33 EAC cell lines was low, cDNA from the high *SPP1*/OPN-expressing cell line H460, a large lung cell carcinoma line, was used as a template for PCR amplification of the individual OPN isoforms (Figure S2A–S2C). PCR products were then resolved in 1% TAE agarose gels, and individual isoforms were cut and purified (Figure S2A). Full-length OPNa, OPNb and OPNc products were ligated (T4 DNA ligase, New England BioLabs) into pcDNA4 (Invitrogen), and pooled, multi-clonal stable cells for each isoform were then cultured and maintained in selective zeocin-containing media (Figure S2B–S2C). LacZ was also cloned into pcDNA4 and selected with the same zeocin-containing media as a control. To achieve expression of the N-terminal integrin only fragments of OPNa, OPNb and OPNc that result from naturally thrombin cleavage at the R/SK site of OPN, isoform-specific PCR cloning products were designed with a stop codon immediately following this cleavage site and then individually cloned into the pcDNA4 vector (Figure S2B). These constructs were designated OPNia, OPNib and OPNic, with “i” indicting the integrin domain-containing only fragment. All expression vector constructs were sequenced to ensure the accuracy of gene inserts. OPN expression levels of each isoform in stable cells were monitored using qRT-PCR (Figure S2D–S2E).

For assays evaluating epigenetic regulation of OPN expression, the demethylating agent 5-aza-2-deoxycytidine (5-Aza) was used at 5 μmol/L for 48 hours before cells were harvested and DNA was extracted.

### Invasion assay

BD Matrigel (Cat. # 354234, BD Bioscience) at 250 μg/ml was thawed on ice and mixed with coating buffer (Tris 10 mM pH 8.0, NaCl 0.7%). The Matrigel mixture (100 μl) was pipetted onto an 8 μm pore insert of a 24-well culture plate and incubated at 37°C for > 2 hours (h). Cells were resuspended in serum-free medium at 0.045 × 10^6^/ml and 0.5 ml cells were seeded on the solidified Matrigel matrix. Bottom chambers were filled with 0.75 ml 10% FBS media. Invasive cells were fixed and stained using the Diff-Quick kit (Fisher Scientific) and counted from five individual locations per insert and quantified using a ratio to matched migrating cells in uncoated wells per the manufacturer's instructions.

### Subcutaneous implantation of OPN stable isoform cells in nude mice

Male nude mice at 6 weeks of age were purchased from Jackson Laboratory (Bar Harbor, Maine) and acclimated for one week before use. All mice were grouped in five or less animals per cage and maintained in the facilities of the Unit for Laboratory Animal Medicine (ULAM) with HEPA-filtered laminar flow rooms under specific pathogen-free conditions. All animal studies were conducted under the guidelines and approved protocols of the University Committee on Use and Care of Animals (UCUCA) of the University of Michigan. Following Lenti-Luc (University of Michigan Vector Core) transduction of all OPN isoform stable cells, 1 × 10^6^ cells were resuspended in 100 μl of 9 μg/μl BD Matrigel and injected subcutaneously into the flanks of nude mice. Mice were scanned with the IVIS Spectrum System (Perkin Elmer, Waltham, MA) 12 min after intraperitoneal administration of 3 luciferin/100 μl DPBS (Regis Technologies, Inc., Morton Grove, IL) at day 2 and day 70 post injection. A fixed region of interest (ROI) was applied to all tumors examined, and the ratio of total flux (photons/second, p/s) was calculated.

### Integrin inhibition in Flo/OPN cells

Cilengitide was purchased from Selleck Chemicals (Houston, TX) and dissolved in PBS at 33 μM. Flo/OPN cells were treated with cilengitide at a final concentration of 1000 nM (600 ng/ml) or vehicle control in 2% FBS media for 6 days, with cilengitide media changes every two days. Cells were allowed to recover in inhibitor-free 10% FBS media for 6 h and then retreated with inhibitor-containing media for an additional 12 h prior to protein isolation.

### Wound healing assay

Flo/OPN stable cells were seeded in triplicate at 0.6 × 10^6^ cells/12-well in 10% FBS DMEM and wounded at 24 h with p20 pipet tips. Cells were then cultured in 2% FBS DMEM containing 0.1% DMSO vehicle control or 100 nM cilengitide in DMSO. Pictures were taken at 0 h, 12 h, 24 h, 48 h, 72 h and 96 h using a microscope-mounted camera (SPOT Idea) and SPOT Software (SPOT Imaging Solutions, Sterling Heights, MI). Wound measurements were collected from 4 different areas per well at each time point for each individual OPN isoform, and migration distances were calculated.

### Gene knockdown using siRNA transfection

*MET* and *ERBB2* gene amplification and expression levels were analyzed in OE33 and Flo cells using real time PCR and RT-PCR (Figure S3D). Primer sequences are listed in Table S1. OE33/OPN stable cells were plated at 0.6 × 10^6^ cells/6-well and transfected with *ON-TARGETplus* siRNA (10 nM, unless otherwise noted) against *MET* (Cat. # L-003156, Dharmacon), *ERBB2* (Cat. # L-003126, Dharmacon) or both (5 nM each) as well as control Non-Targeting siRNA (Cat. # D-001210, Dharmacon) using Lipofectamine RNAiMAX (Invitrogen) at a final concentration of 1 μl/ml according to the manufacturers' instructions. Cells were cultured for 96 h with an addition of fresh siRNA at 48 h. Mock transfections were performed using Lipofectamine RNAiMAX alone. Cells were then fixed and stained with Diff-Quick solutions per the manufacturer's instructions.

### Cell adhesion assays

BD Matrigel (above mentioned), containing > 60% laminin, was thawed on ice and mixed with DPBS (GIBCO) to a final concentration of 18 μg/ml (laminin ≅ 10.8 μg/ml). Tissue culture plates (96-well) were coated with 100 μl/well of Matrigel/DPBS mixture and DPBS was added to uncoated wells as controls. The plates were kept at room temperature for 60 min followed by aspiration of the supernatant DPBS solution. Blocking solution (200 μl), containing 10 mg/ml BSA/DPBS that had been 0.22 μm filtered and heat denatured at 85°C for 10 min, was added to each well and incubated at room temperature for 30 min. Blocking solution was then aspirated and wells were washed once with 100 μl DPBS. Single cell suspensions (0.01 × 10^6^ cells/well) in pre-gassed DMEM/HEPES (GIBCO) were plated in laminin-coated or uncoated 96-well plates and incubated at 37°C, 5% CO_2_ for 30 min. Cell suspensions were then aspirated and 100 μl of 5% glutaraldehyde (w/v) was added per well and incubated at room temperature for 30 min followed by three gentle washes with 100 μl DPBS. Plates were then stained with Diff-Quick solution and cells were counted per FOV (field of view). Triplicate wells for each isoform and its uncoated control were analyzed in each experiment.

### Anchorage-independent growth and clonogenic assays

Powdered DMEM and RPMI (Sigma-Aldrich) were purchased to make 2x complete media with 20% FBS with or without 2x1000 nM of cilengitide. Both base-agar (1.3%) and top-agar (0.7%) were also made at 2x concentrations. Following equilibration in a 40°C water bath, base agar was mixed with 2x complete media with or without 2x1000 nM cilengitide in a 1:1 ratio, cast in triplicate wells of 12-well plates, and allowed to solidify at room temperature (RT) for > 1 h. Similarly, top-agar and 2x media were mixed with single-cell suspensions of Flo/OPN (7000 cells/well) or OE33/OPN (5000 cells/well) stable cells, added to the solidified base-agar, and allowed to solidify for 30 min at RT. Plates were overlaid with complete media (200 μl) and incubated at 37°C/5% CO_2_. A diluted aliquot of each cell group was plated in regular culture dishes (150 cells/well in 6-well plates) to monitor cell counting and viability as well as the efficacy of 1000 nM cilengitide, the pre-determined IC_50_ dose for Flo/LacZ cells. Agar-plated cells and clonogenic-assayed cells were incubated at 37°C/5% CO_2_ for 12 days (clonogenic assays) or 14-30 days (soft agar assays). Colonies were then stained with 0.05% crystal violet in 50% ethanol. Agar plate images were acquired using an Olympus DP-71 digital camera and Leica MXFL III stereo fluorescent microscope (Leica Microsystems). Colony numbers were quantitated using ImageJ software (http://rsb.info.nih.gov/ij/).

### Statistical analysis

Graphs and Pearson correlation coefficients were generated using GraphPad Prism5 software (La Jolla, CA), and *p*-values were determined by log-rank test, *t*-test, and one-way or two-way ANOVA as appropriate.

## SUPPLEMENTARY MATERIALS AND METHODS


