# Supplementary material for: Osteopontin (OPN/SPP1) isoforms collectively enhance tumor cell invasion and dissemination in esophageal adenocarcinoma
Source: Oncotarget. 2015 Jun 1;6(26):22239–57. doi: 10.18632/oncotarget.4161 (PMC4673160; doi:10.18632/oncotarget.4161)
Supplement: Supplementary file 1 [file oncotarget-06-22239-s001.pdf]

SUPPLEMENTARY MATERIALS AND METHODS

SNP Array experiments and analysis

SNP arrays were performed as previously described [1, 2]. Briefly, DNA copy numbers of 73 EACs were assessed using the Genome-Wide Human Sty I 250K SNP Array (Affymetrix) (GSE36460). Copy number analyses were performed using a log2 copy number ratio, with +0.848 designated as amplification and -0.737 designated as deletion. Genomic positions were mapped in the hg18 genome build. SNP data were visualized using the software IGV 1.3.1 (Integrative Genomics Viewer, http://www.broadinstitute.org/igv).

Western blot analysis

Protein samples (10–40 µg) were resolved on Tris-Glycine gels (Invitrogen) and blotted to PVDF membranes (Millipore). Membranes were blocked with either 5% milk (Bio-Rad) or 5% BSA (Sigma-Aldrich) in 1xTBST per the primary antibody manufacturers' recommendations. Primary antibodies Vimentin (#5741, Cell Signaling), E-cadherin (#3195, Cell Signaling), β-catenin (# 610153, BD Biosciences) and GAPDH (#5174, Cell Signaling) were diluted 1:1000 and were hybridized at 4°C overnight. Goat anti-rabbit (Cat.# PI-4100; Vector Laboratories, Inc.) or anti-mouse (Cat.# 1010–05, Southern Biotech) HRP-conjugated secondary antibodies were diluted 1:10, 000 and were hybridized for 1 hour at room temperature. Western blot detection was performed using GE Amersham ECL Prime (RPN2232). Membranes were stripped for re-hybridization using ReBlot Plus Strong Antibody Stripping Solution (Millipore) and were reprobed with GAPDH or β-actin.

Clonogenic and wound healing assays using OPN-conditioned media

OPN-null OE19 cells were plated at 0.001×10^6 cells/60 mm plate for clonogenic assays and 0.4×10^6 cells/24-well for wound healing assays. Individual Flo/OPN isoform cells were seeded at 4×10^6 cells/100 mm plate in 10% FBS RPMI medium without zeocin selection and allowed to attach for 24 hours. Forty-eight hour Flo/OPN isoform-conditioned media was collected and mixed with fresh 10% FBS media at a 3:1 ratio and then was added to OE19 cell cultures every 48 hours. In clonogenic assays, cells were fixed with Diff-Quick fixation solution at 16 days per the manufacturer's instructions. Wound healing assays were imaged as previously described for up to 9 days.

REFERENCES

1. Bass AJ, Watanabe H, Mermel CH, Yu S, Perner S, Verhaak RG, Kim SY, Wardwell L, Tamayo P, Gat-Viks I, Ramos AH, Woo MS, Weir BA, Getz G, Beroukhim R, O'Kelly M, et al. SOX2 is an amplified lineage-survival oncogene in lung and esophageal squamous cell carcinomas. Nat Genet. 2009; 41:1238–1242.
2. Dulak AM, Schumacher SE, van Lieshout J, Imamura Y, Fox C, Shim B, Ramos AH, Saksena G, Baca SC, Baselga J, Tabernero J, Barretina J, Enzinger PC, Corso G, Roviello F, Lin L, et al. Gastrointestinal adenocarcinomas of the esophagus, stomach, and colon exhibit distinct patterns of genome instability and oncogenesis. Cancer Res. 2012; 72:4383–4393.

SUPPLEMENTARY FIGURES AND TABLE

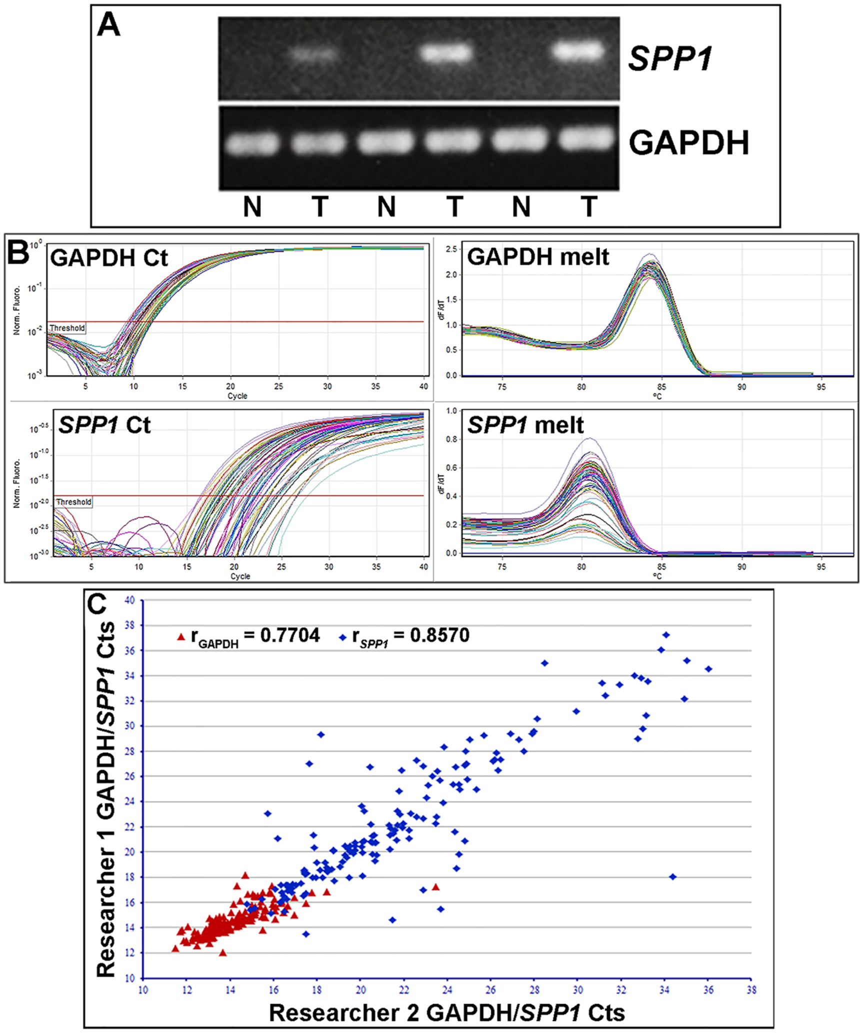

Supplementary Figure S1: Representative images of qRT-PCR quality monitoring. A. Specificity of the *SPP1* primer pair was elucidated using standard PCR and 1% agarose gel electrophoresis. **B.** Ct and melt curves were analyzed for *SPP1* and *GAPDH* real-time PCR amplifications in EAC patient samples. **C.** qRT-PCR was performed by two individual researchers and the correlation of Ct values was analyzed.

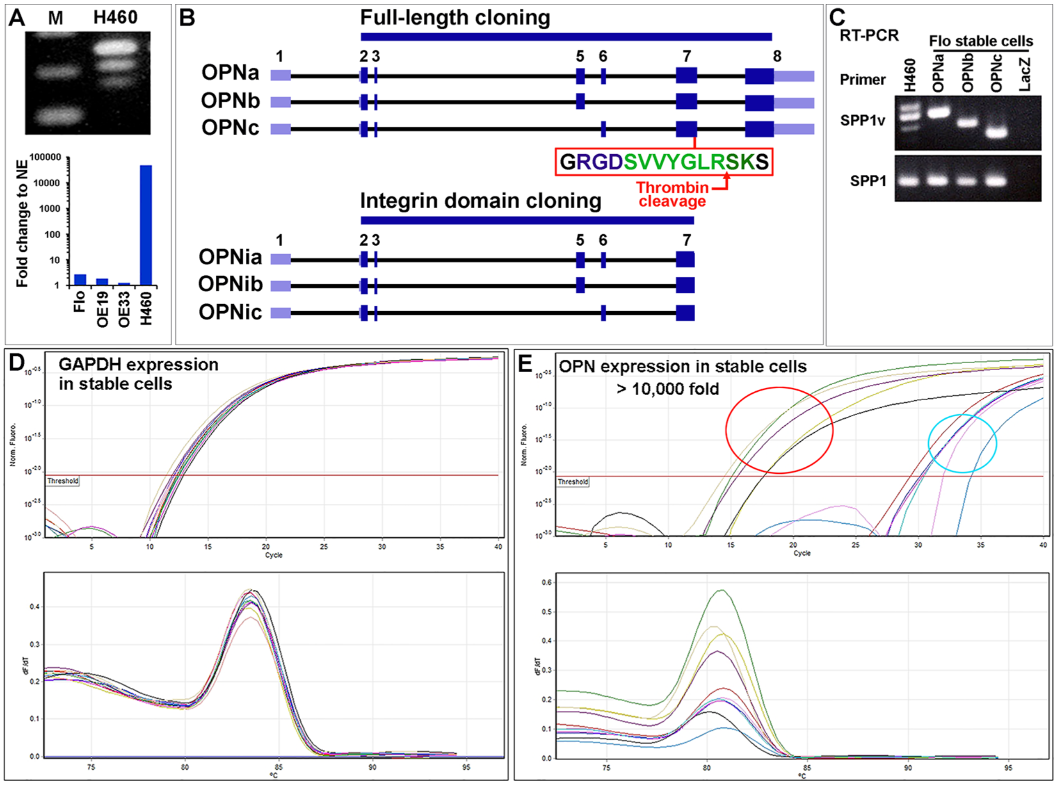

Supplementary Figure S2: Cloning strategy of SPP1/OPN isoforms and cleaved isoforms. A. Cloning fragments of OPN isoforms a, b, and c were PCR-generated using cDNA templates from an OPN high-expressing cell line, H460, and the splicing variable regions were analyzed (primers were listed in Table S1). Endogenous OPN expression in EAC cell lines and the H460 cell line was measured and analyzed using qRT-PCR. (NE, normal esophageal sample) **B.** Schematic of full-length isoform cloning and isoform integrin-domain only cloning. The thrombin-cleavage site "R/SK" was used to design primers for N-terminal integrin-domain constructs (OPNia, OPNib, and OPNic) that did not contain the CD44 domain. (light blue, non-coding exon sequences; blue, coding exon sequences) **C.** Established Flo/OPN stable cells were validated by PCR and confirmed by sequencing (primer pairs listed as SPP1 and SPP1v in Table S1). **D–E.** Real-time RT-PCR was used to analyze the ectopic OPN expression levels of individual isoform stable cells (red-circled Ct curves) as compared to endogenous OPN expression levels in Flo and OE33 cells (blue-circled Ct curves).

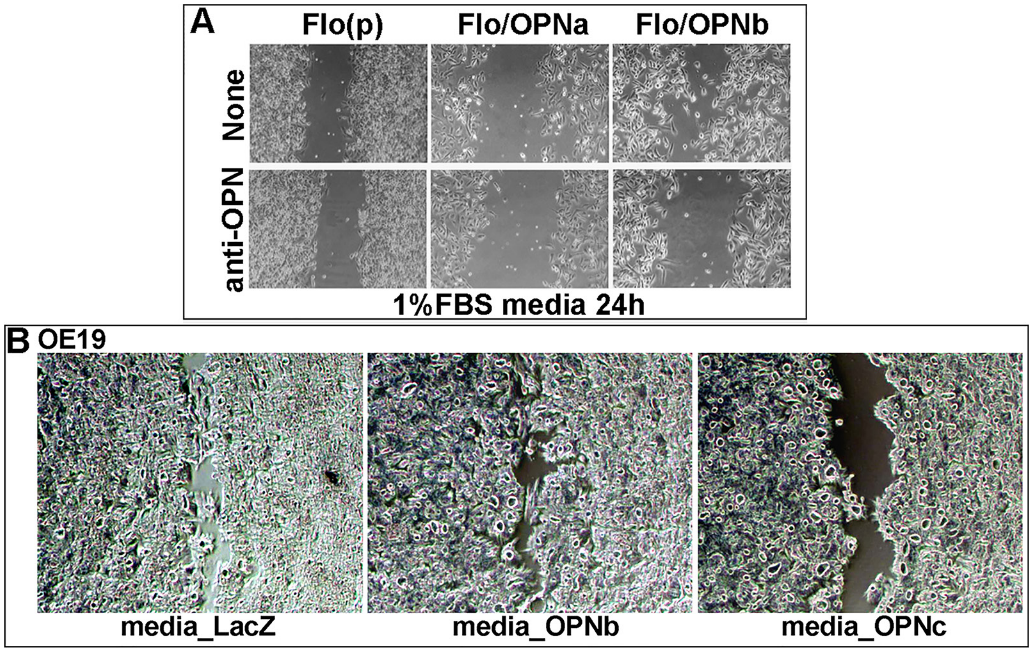

Supplementary Figure S3: Cell migration and detachment assays in OPN isoform stable cells. A. Wound healing assays using Flo/OPN stable cells showed that addition of OPN antibody attenuated the enhanced migration of OPNb cells at 24 hours post wounding. (Flo(p), parental Flo cells) **B.** OPN-null OE19 cells migrated slower when cultured with Flo/OPNc than with Flo/OPNb donor-media, which was added to the wounded OE19 cells every 48 hours. Images shown were taken on day 9.

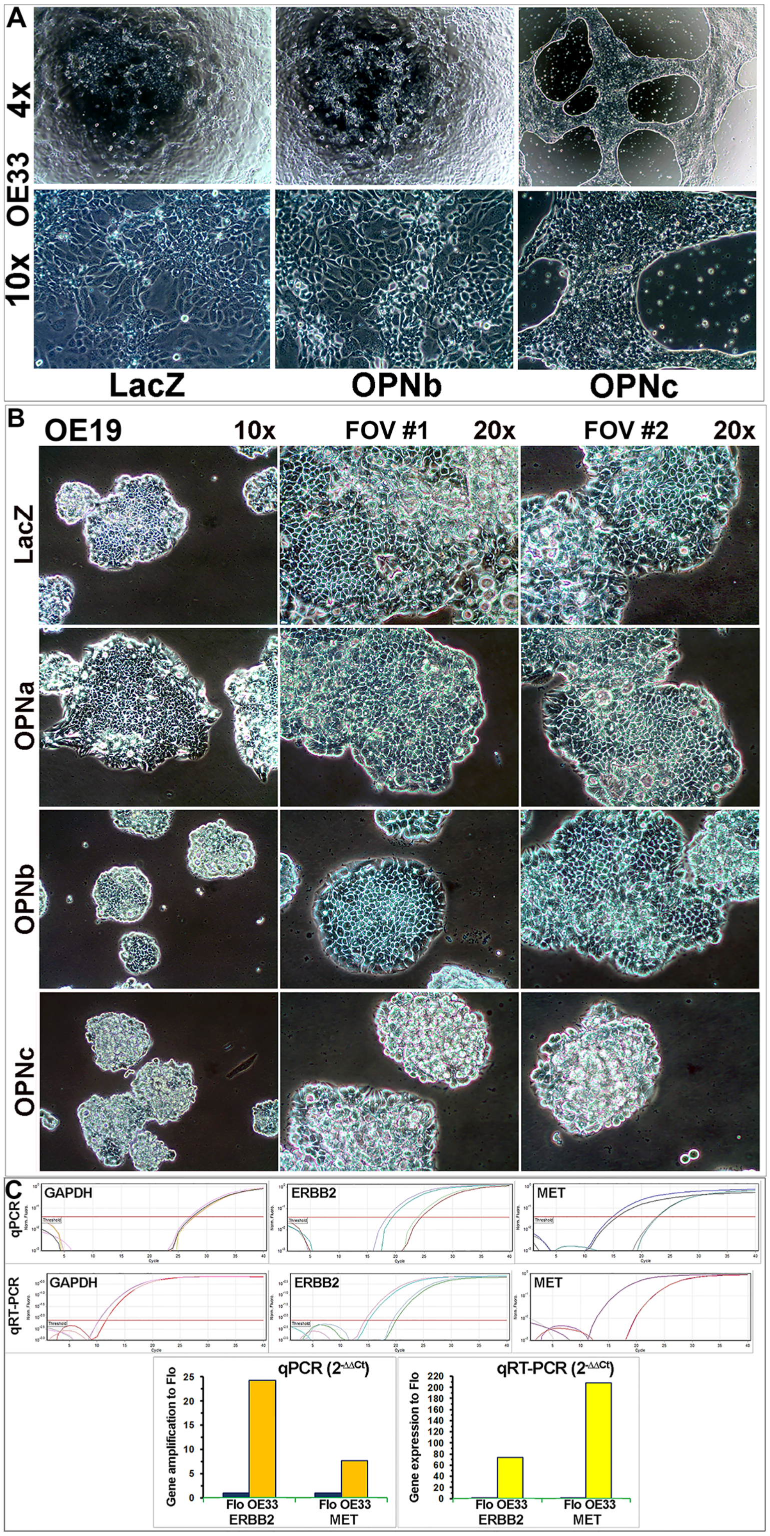

Supplementary Figure S4: OPN isoform-induced growth phenotypes in EAC cells and MET/ERBB2 gene amplification in OE33 cells. A. OE33/OPN stable cells (0.25×10^6 cells/ml) were seeded and allowed to grow to confluence. Images shown were taken 6 days post-seeding. Detachment of OPNc-expressing cells was significantly elevated as compared to OPNb-expressing and LacZ control cells. **B.** OPN isoform-donor media changes the growth pattern of OE19 cells. OPN-null OE19 cells (1, 000 cells/60 mm dish) were cultured in the conditioned-media of individual Flo/OPN isoform-expressing stable cells that had been collected and added to OE19 cells every 48 hours for 16 days. OPNc-media promoted more multi-layer spherical-shape growth as compared to cells cultures with OPNa-, OPNb- or LacZ-media, which predominantly resulted in single-layer growth. **C.** Gene amplification and overexpression of *MET* and *ERBB2* was observed in OE33 cells as compared to Flo cells using real-time PCR and RT-PCR (duplicate reactions were done).

Supplementary Table S1: Primers used in the present study*

Name	Forward	Reverse	Length (bp)	Use/position	Application
SPP1	TGGCCGAGGTGATAGT GTGGTTTA	AACGGGGATGG CCTTGTATGC	151	exon 7–8	qRT-PCR
SPP1v	ACAGGCTGATTCTGG AAGTTCTGA	GTCAATGGAGT CCTGGCTGTCC	253 a, 211 b, 172 c	exon 3–7	^{32}P-labeled RT-PCR; cloning insert examination
SPP1iso4	ACGCCGACCAAGGA AAACTCAC	GTCATGGCTTT CGTTGGACTTACTT	152	exon 1–7	Size selectable PCR for isoform OPN4
SPP1iso5	GATGTACCTACCCCTCC ACAACAGATGA	AGTGACCCCCA AGGCAGCTCTATTT	148	exon 4	Exon 4 specific PCR for isoform OPN5
SPP1g	TGATGGCCGAGGTGAT AGTGTGG	AAAACCGCCA AGCAGAAAAAGAT	245	Genomic DNA	^{32}P-labeled qG-PCR for SPP1 copy number examination
SPP1cloning	AGAATTGCAGTGATT TGCTTTTGC	CTCCTTTTAATTG ACCTCAGAAGATGC	Varied by isoforms	full-length	Full-length TOPO cloning
SPP1itg-cloning	HindIII-[1]AGCGCAGAGG CTTGGGGCAG	XbaI-[2]**TTA**TTTTG ACCTCAGTCCATAAAC	Varied by isoforms	integrin domains	thrombin cleaved site cloning
GAPDHint	TCTTCCCACCCGCC CCAGTC	TGCCCAACACC CCCAGTCATAC	296	GAPDH intron	^{32}P-labeled qG-PCR for GAPDH copy number examination
GAPDH5′rt	TCGACAGTCAGCC GCATCTTCTTT	GCCAGCATCGC CCCACTTGA	331	exon 1–5	^{32}P-labeled qRT-PCR for GAPDH expression examination
METg	CTGATGGCCACCTG TATGACTTAG	GATGACCGGAT CCCTTTTGTAA	128	MET intron 1	MET amplification
METrt	GAGCCCCACCTTAT CCTGACG	GGGCGCATTT CGGCTTTAG	145	exon 20–21	MET overexpression
ERBB2g	GGGAGAACCACC GCATTGACTT	GCGCCCAGG CTCTTGACACT	142	[3]ERBB2 intron 15	ERBB2 amplification
ERBB2rt	GCCCCATCAACTG CACCCACTC	GACCACCCCC AAGACCACGACC	134	[3]exon 15–17	ERBB2 overexpression

*All real-time primers for GAPDH and ACTB were published previously in reference 98.
[1]Vector pcDNA4 sequence;
[2]Bold, a stop codon;
[3]Intron and exons were positioned in AC087491 and NM_004448.
